# Repression of KIAA1199 attenuates Wnt-signalling and decreases the proliferation of colon cancer cells

**DOI:** 10.1038/bjc.2011.268

**Published:** 2011-07-19

**Authors:** K Birkenkamp-Demtroder, A Maghnouj, F Mansilla, K Thorsen, C L Andersen, B Øster, S Hahn, T F Ørntoft

**Affiliations:** 1 Department of Molecular Medicine MOMA, Center for Molecular Clinical Cancer Research, Aarhus University Hospital/Skejby, DK-8200 Aarhus N, Denmark; 2 Center for Clinical Research (ZKF), Molecular GI-Oncology (MGO), Department of Internal Medicine, Knappschaftskrankenhaus, Ruhr-University Bochum, D-44780 Bochum, Germany

**Keywords:** Wnt/*β-*catenin signalling, tumour marker, colorectal cancer, proliferation, cell cycle

## Abstract

**Background:**

The KIAA1199 transcript is upregulated in colon adenomas and downregulated upon *β*-catenin knockdown.

**Methods:**

Transcript profiling was performed on >500 colon biopsies, methylation profiling data were compared with transcript data. Immunohistochemistry assessed KIAA1199 protein expression in 270 stage II/III tumours (>3 years follow-up). The effects of stable KIAA1199 knockdown in SW480 cells (three different constructs) were studied using transcriptional profiling, proliferation and protein analysis.

**Results:**

The KIAA1199 transcript was strongly upregulated in 95% of adenocarcinomas. Absent expression in normal mucosa correlated with KIAA1199 promotor methylation. Nuclear and cytoplasmic KIAA1199 protein expression was identified in colon adenocarcinomas and other types of cancers. A subpopulation of patients with tumours strongly expressing KIAA1199 in the nucleus showed a better outcome with regard to recurrence as lung or liver metastases. The KIAA1199 knockdown affected the cell cycle and the Wnt-signalling pathway. Reduced cellular proliferation and decreased KI67, phosphorylated retinoblastoma, *β*-catenin and ASCL2 protein expression supported these findings. Eighteen Wnt-signalling genes differentially expressed upon KIAA1199 knockdown correlated with the KIAA1199 expression profile in clinical specimens.

**Conclusion:**

The KIAA1199 knockdown attenuates the effects of the Wnt/*β-*catenin signalling and it may thus be regarded as a regulatory part of this pathway.

Colorectal cancers (CRC) account for ∼10% of the total worldwide cancer cases, with an overall 5 years survival of around 50% (Boyle and Levin, 2008). Early diagnosis and improved treatment of CRC requires the identification of new biomarkers as well as insights into the molecular mechanisms of human carcinogenesis.

Most CRCs are caused by aberrant Wnt-signalling, which in 70–80% of cases are rooted in mutational inactivation of the tumour-suppressor gene *APC* (Bienz and Clevers, 2000). This leads to stabilisation and nuclear translocation of the proto-oncogene *β*-catenin. *β*-catenin has a crucial role in cell–cell adhesion and is part of the transcription factor complex *β*-catenin/TCF, recruiting numerous co-regulators to achieve activation or inhibition of target gene promoters in response to Wnt-signalling (Gregorieff and Clevers, 2005). Subsequent to APC inactivation follow mutations in oncogenes-like K-Ras and tumour-suppressor genes like *TP53* (Kinzler and Vogelstein, 1996). Additional genetic changes may appear in CRC, for example, the apparent loss of the response to transforming-growth factor-*β* (TGF-*β*) (Polyak, 1996). The TGF-*β*-signalling occurs through Smads-2/3/4, translocating to the nucleus to regulate transcription. However, Smad4 also has a TGF-*β*-independent function as a tumour suppressor cooperating with *β*-catenin/Lef to regulate target gene expression (Tian *et al*, 2009).

In a previous transcript profiling study on a small sample set, we identified the KIAA1199 transcript to be strongly upregulated in colon adenocarcinomas stage I-IV compared with normal mucosa (Birkenkamp-Demtroder *et al*, 2002). The KIAA1199 was also found to be upregulated in colon adenomas (Sabates-Bellver *et al*, 2007). Induction of TCF4 proteins or siRNA-mediated knockdown of *β*-catenin in LS174 colon cells decreased KIAA1199 transcript expression (Sabates-Bellver *et al*, 2007). The TCF4 protein *(TCF7L2)* is a transcription factor involved in the Wnt/*β-*catenin-signalling pathway and findings of genome-wide TCF4 ChIP-on-chip analyses indicate that the KIAA1199 locus is surrounded by four TCF4-binding regions (Sabates-Bellver *et al*, 2007), suggesting an involvement of KIAA1199 in the Wnt/*β*-catenin-signalling pathway.

The *KIAA1199* gene (Hs.459088, chr.15q25.1, *TMEM2 L*) encodes a 150 kDa protein and was originally described as an inner ear protein (Abe *et al*, 2003). KIAA1199 has been associated with cancer tissues: high KIAA1199 transcript levels were found in mortal but not immortal renal cell carcinoma cells (Michishita *et al*, 2006) whereas low KIAA1199 expression in gastric cancers was related to significant better outcome of the patients (Matsuzaki *et al*, 2009).

Despite previous studies indicating an upregulation of KIAA1199 in neoplasic CRC tissues, this has not been confirmed in a large set of samples, neither with regard to transcript or protein levels, nor with regard to clinical outcome. Moreover, KIAA1199's putative cellular functions and pathway interactions have not been reported previously.

The aim of this study was to characterise the KIAA1199 transcript and protein expression in colorectal adenocarcinomas, the correlation of KIAA1199 expression with patient outcome and the identification of KIAA1199-related pathways to elucidate the role of KIAA1199 in CRC.

## Materials and methods

### Tissue samples

Data from four different tissue sample sets were used in this study: set 1: U133A-arrays, *n*=122 (Kruhoffer *et al*, 2005); set 2: U133plus2.0-arrays, *n*=389 (Andersen *et al*, 2009); set 3: Exon1.0ST-arrays, *n*=40 (Øster *et al*, 2011); set 4: Exon1.0ST-arrays, *n*=27, (Thorsen *et al*, 2008)). Informed written consent was obtained from all patients and the studies were approved by the Central Denmark Region Committees on Biomedical Research Ethics.

Methylation profiling on 40-microdissected colon tissue samples were analysed on Infinium HumanMethylation27 BeadChips (Illumina, San Diego, CA, USA) as described in (Øster *et al*, 2011). In brief, DNA extracted from fresh frozen tissue samples was bisulfite modified, whole genome amplified and hybridised to Beadchips.

Colon cell lines obtained from American Type Culture Collection (ATCC-LGC standards, Borås, Sweden) were re-authenticated via STR analysis (Masters *et al*, 2001) using the Cell-ID-system (G9500, Promega, Nacka, Sweden). No mycoplasma contamination was detected using nested PCR. RNA was extracted using GenElute Mammalian Total RNA Miniprep Kit (RTN350, Sigma-Aldrich, Brøndby, Denmark). RNA (RIN⩾9.9) was analysed on (a) U133plus2.0 GeneChips; comparison analysis was performed using MAS5.0 software (Affymetrix, Santa Clara, CA, USA). Probes accompanied by an Inc/Dec call and a log_2_ ratio ∣>0.5∣ were included, but excluded when listed as ‘absent’ or (b) ExonST1.0arrays; data were quantile-normalized with ExonRMA16-algorithm and data analysis was performed using GeneSpring GX10-software (Agilent, Hørsholm, Denmark). Genes were annotated using the Affymetrix NETAFFX annotation (NCBI Build 36.1, netaffx-build=28, Santa Clara, CA, USA).

### Antibody synthesis and cloning of KIAA1199

Polyclonal rabbit anti-KIAA1199 antibodies were raised against peptide CARYSPHQDADPLKPR (amino-acids 772–776 in Q8WUJ3, Eurogentec, Seraing, Belgium). Antibody specificity of the affinity-purified antibody was confirmed by ELISA (Eurogentec), western blotting using recombinant V5-His- or GFP-C-terminal-tagged KIAA1199 proteins (full length and splice variant), cloned to a previously published procedure (Birkenkamp-Demtroder *et al*, 2007) as well as immunohistochemistry on formalin-fixed and paraffin embedded cells (FFPE) as described in the Supplementary data. Western Blotting was performed on 4–12% gradient gels (Olesen *et al*, 2005). Loading control anti-*β*-actin monoclonal antibody 0.05 *μ*g ml^−1^ (#A-1978, clone AC-15, Sigma-Aldrich) (Marker ‘All Blue’ (Bio-Rad, Copenhagen, Denmark)).

### FFPE cells

The FFPE cells 80% confluent cells were harvested by scraping, washed with 10 ml HANK's buffer (formaldehyde solution 4%, pH 7) and pelleted at 1200 r.p.m. for 5 min. The supernatant was removed and 5 ml 10% formalin were added and incubated at 4°C overnight. The supernatant was removed and the pellet was dehydrated by adding 2 × 5 ml 70% EtOH (Kemethyl A/S, Køge, Denmark), 2 × 96% EtOH, 3 × 99% EtOH and 3 × Xylen (Merck, Hellerup, Denmark), incubation 5 min each. Shandon histoplast paraffin (Hounisen, Risskov, Denmark) was added and the pellet was incubated overnight at 60°C. The pellet was transferred to a paraffin cutting tray, paraffin embedded and 8 *μ*m thick sections were cut and immunostained.

Immunohistochemistry was performed on FFPE specimens as previously published (Birkenkamp-Demtroder *et al*, 2007), using 4 *μ*m tissue sections from the colon biopsies and 8 *μ*m sections from FFPE cell culture cells. Tissue slides were scanned with a Nanozoomer (Hamamatsu, Herrsching am Ammersee, Germany) and cores were analysed by VIS-TMA-software (Visiopharm, Hørsholm, Denmark).

### Tissue microarray analysis

We analysed KIAA1199 protein expression on a UICC stage I-IV CRC TMA (COCA912-5-OL, BioCat, Heidelberg, Germany), a human various tumours and corresponding normal specimens TMA with 94 cases (T8235713-5-BC, BioCat, previously used in (Mansilla *et al*, 2009)) as well as a TMA with a total of 438 stage II/III samples and detailed clinical data (MOMA). Recurrence was defined as distant recurrence. Patients with other cancers than CRC, with multiple CRC, with local recurrence or recurrence in the form of carcinosis as well as with <3 years of follow-up were excluded from the study, resulting in 270 stage II samples with 3 years of follow-up. Scoring was independently done by two experienced observers (KBD, CLA). Weighted kappa statistics showed a good interobserver agreement for cytoplasmic (*κ*=0.8806) and nuclear KIAA1199 expression (*κ*=0.6077).

The shRNA-vector preparation and lentivirus production was performed according to (Stewart *et al*, 2003) generating five KIAA1199-specific knockdown constructs. Quantitative RT-PCR was used to validate efficient knockdown and data were normalised against GAPDH, HPRT1 and PPIA (see Supplementary data).

### Immunofluorescence microscopy

Cells seeded on glass chamber slides (#178599, Nunc, Roskilde, Denmark) were fixed, stained and mounted as previously described (Birkenkamp-Demtroder *et al*, 2007). Primary antibodies are as follows: monospecific rabbit anti-KIAA1199 antibody (4–6 *μ*g ml^−1^); anti-KI67 (1 : 400, DAKOcytomation, Copenhagen, Denmark), anti-*β*-catenin (1 : 400 IF, BDBiosciences, Brøndby, Denmark), anti-V5-tag (SV5-Pk1) (1 : 3000, ab27671, Abcam, Cambridge, UK), anti-RB-pS^795^ (1 : 250, monoclonal anti-phosphoretinoblastoma (RB) (pS795) clone RB-10, R6878, Sigma-Aldrich) and anti-ASCL2 7E2-supernatants (1 : 5, (Van der Flier *et al*, 2009)). The specificity of the ASCL2 antibody was proven on western blots of extracts from ASCL2 overexpressing cells, by ChIP-analyses and inducible ASCL2 RNAi in LS174 colon cancer cells (personal communication Laurens Van der Flier). Secondary antibodies are as follows: AlexaFlour 488 goat anti-mouse or goat anti-rabbit IgG highly cross-adsorbed (1 : 2000, Molecular Probes, Invitrogen, Taastrup, Denmark). Nuclei were counterstained with DAPI or Hoechst and slides were mounted with fluorescence mounting medium (DAKOcytomation).

### Ingenuity pathway analysis

Data below background noise (threshold log2<4.0) were excluded from analyses (IPA version 8.5, Redwood City, CA, USA). Expression values were normalised around zero. Normalised ratios given as (−INF, −1) and (1, +INF) were submitted to ingenuity pathway analysis (IPA).

Real time cell analyses of proliferation, adhesion and migration were performed in triplicates on xCELLigence instruments (Roche, Hvidovre, Denmark).

Cell proliferation was assessed by an MTT-assay (3-(4,5-dimethylthiazol-2-yl)2,5-diphenyltetrazolium bromide, Roche) on a Synergy-HT Microplate-Reader (Biotek, Bad Friedrichshall, Germany), readout at 540 nm, absorbance at 692 nm was used as reference.

Cell adhesion was analysed as previously described (Weinacker *et al*, 1994). Briefly, 2000–4000 cells in a total volume of 50 *μ*l volume (*n*=10) were incubated on 96-well-plates coated with fibronectin (F0895, Sigma-Aldrich), readout at 550 nm.

Statistical analysis was performed using STATA 10 (Statacorp, College Station, TX, USA). Transcript values were expressed as median log_2_ ±s.d. A two-tailed Student's *t*-test or log-rank test for survival estimates was applied and *P*-values <0.05 were considered as statistically significant.

## Results

### KIAA1199 is strongly upregulated in most tumours of the large intestine independent of their location

**Figure 1:**
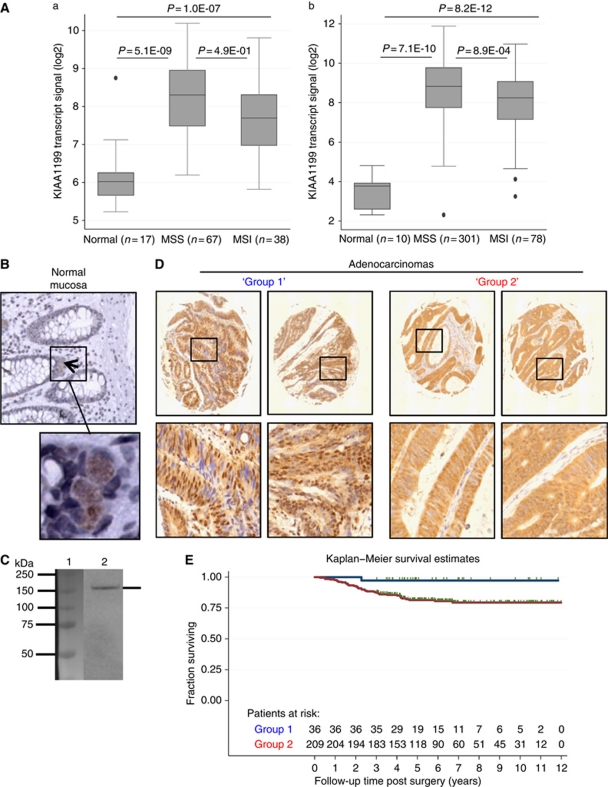
The KIAA1199 transcript and protein expression and correlation to clinical outcome. (**A**) The KIAA1199 transcript profiling of 511 colon samples (normal mucosas, MSS microsatellite stable and MSI instable and adenocarcinomas). (a) The U133A GeneChips (*n*=122); (b) U133plus2.0 GeneChips (*n*=389); (details shown in Supplementary Figure 1). (**B**) Normal colon mucosa showed a very weak expression in the cytoplasm of single cells located in the bottom of the crypts. (**C**) Western Blot of SW480 cells overexpressing KIAA1199-V5-HIS protein (157 kDa). Lane 1 – Marker Bio-Rad All Blue; lane 2 – the monospecific rabbit polyclonal anti-KIAA1199 antibody was specific for KIAA1199 (specificity is shown in Supplementary Figures 2a and b). (**D**) Immunohistochemical analysis of 270 stage II adenocarcinomas (>3 years follow-up) using the monospecific anti-KIAA1199 antibody: group 1 showed strong nuclear but weak cytoplasmic KIAA1199 expression, group 2 showed moderate to strong cytoplasmic KIAA1199 expression, no nuclear expression. (**E**) In all 245 of the 270 samples were informative (118 female, 127 male). Patients without disease recurrence (*n*=205, median follow-up time 2198 days, range 1099–4368); patients with metastatic recurrence (*n*=40, median time to recurrence 830 days, range 95–2449). Kaplan–Meier survival estimates for disease-free survival showed that patients with group 1 tumours (blue line) had a significant (log-rank test *P*-value 0.02) better outcome than group 2 patients (red line). Green ticks: patients censored at death or end of follow-up.

Microarray transcript profiling using U133A (set1) and U133plus2.0 GeneChips (set 2) was performed on 511 sporadic CRC tissue samples obtained from multiple institutions in four countries (Kruhoffer *et al*, 2005), (Andersen *et al*, 2009). The KIAA1199 transcript was significantly upregulated (*P*<1E-07) in MSS and MSI colon adenocarcinomas compared with normal mucosa (Figure 1a), and was strongly expressed in >95% of colon adenocarcinomas (log_2_>7; Supplementary Figures 1a and b). The KIAA1199 transcript level was significantly higher in MSS than MSI tumours (*P*=8.9E-04). No difference was seen between samples from proximal versus distal colon or between stage II versus stage III tumours (data not shown), indicating an almost obligatory high expression of the KIAA1199 transcript in CRC. We identified a slightly, but significantly lower expression in tumours from stage II–III patients with recurrence (*n*=54, median log_2_=8.6) compared with those without recurrence (*n*=241, median log2=8.8, *P*=0.03). Immunohistochemical analysis (IHC) of KIAA1199 protein expression was performed using a monospecific, affinity-purified antibody. The antibody's specificity was proven by western blotting (Figure 1c, Supplementary Figure 2a) as well as by immunohistochemical analysis of FFPE cells (Supplementary Figure 2b).

The IHC analysis revealed a strong KIAA1199 protein expression in the nucleus and/or cytoplasm of the majority of UICC stage I–IV adenocarcinomas (*n*=40). No relationship between staining intensity and disease stage was found (data not shown). In addition, other types of cancers from, for example, breast, kidney, lung and stomach also showed upregulated KIAA1199 protein levels (data not shown). In normal colon mucosa (*n*=10), KIAA1199 was either not or only very weakly expressed in the cytoplasm of single cells located in the proliferative compartment at the bottom of the crypts (Figure 1b).

To study the relation to clinical outcome of CRC, we used a TMA with 270 stage II colorectal adenocarcinomas with at least 3 years of follow-up, as stage II cancers include a small group of about 20% of patients that will progress and should be identified for chemotherapeutic treatment. Remarkably, a small group of patients with tumours strongly expressing KIAA1199 in the nucleus accompanied by weak cytoplasmic expression (Figure 1d) had a significantly (log-rank test *P*-value 0.02) better outcome compared with those with strong cytoplasmic, or nuclear and cytoplasmic KIAA1199 expression using Kaplan–Meier survival estimates of disease-free survival (Figure 1e).

To explore if the KIAA1199 transcript levels are regulated by DNA methylation, we investigated DNA promoter methylation in normal colon mucosas (*n*=6), adenomas (*n*=6) and adenocarcinomas (*n*=28), using Infinium HumanMethylation27 BeadChips (Illumina). The analysis showed that all six normal mucosa samples were methylated at target position cg20828084 (median avg beta=0.63, see details in Øster *et al*, 2011). This position was found to be less methylated in the majority of the stage II–III adenocarcinomas with MSI (5/6) or MSS status (23/24) (median avg *β*=0.47). Parallel expression profiling of these samples (set 3) showed that the KIAA1199 transcript was absent in normal and present in cancer (Supplementary Figure 1c), indicating that KIAA1199 may be regulated, at least partly, by CpG island methylation. Clinical correlation and KIAA1199 upregulation in the majority of colon adenocarcinomas prompted us to study the effects of KIAA1199 dysregulation *in vitro*.

### KIAA1199 upregulation or knockdown in several colon cell lines alter pathway-specific transcripts and the cellular phenotype

We overexpressed V5-His-tagged-KIAA1199 in HCT116 and SW480 cell lines (protein expression and localisation are shown in Supplementary Figures 2c and d) and performed transcript profiling on U133plus2.0 arrays compared with control cells transfected with an empty vector. A 28-fold upregulation of KIAA1199 in HCT116 cells resulted in 306 deregulated genes, whereas an 80-fold upregulation in SW480 cells resulted in 1304 genes (log_2_ >∣0.5∣) being differentially expressed. Common to both cell lines were 33 genes. The IPA showed that molecular and cellular functions of the cell cycle (135 molecules, *P*=3.57E-12), cell death (224, *P*=4.72E-10), cell division process (*P*=3.65E-11), cellular growth and proliferation (236, 7.45E-07), cellular movement (66, 4.59E-06), cell morphology (133, 2.51E-06), as well as DNA replication and repair (67, 8.35E-06) were affected. Moreover, canonical pathways such as apoptosis, Ephrin-receptor- and Wnt/*β*-catenin-signalling were also affected (*P*<0.05).

**Figure 2:**
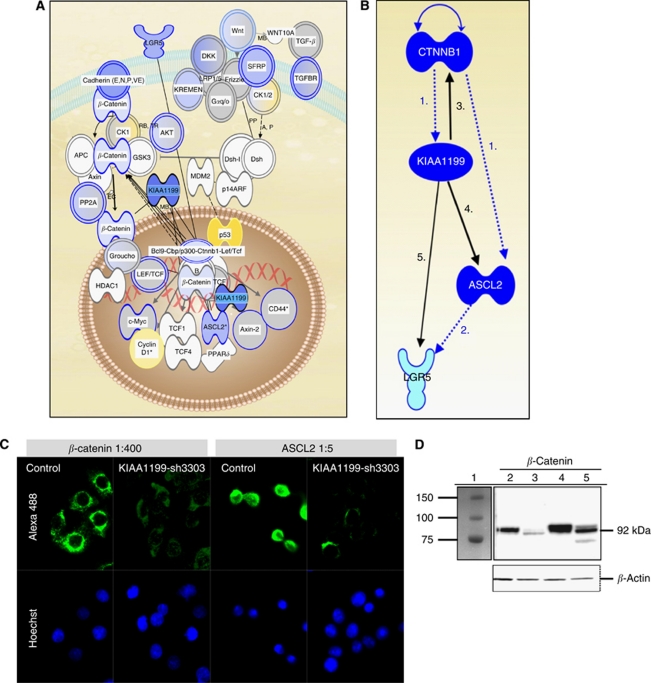
KIAA1199 knockdown affects the Wnt/*β*-catenin signalling pathway. (**A**) The IPA showed that a number of genes involved in canonical Wnt/*β-*catenin signalling were up (yellow) or downregulated (blue) upon KIAA1199 knockdown. (**B**) Summary of relations between KIAA1199, *β*-catenin, ASCL2 and LGR5: (1) knockdown from *β*-catenin shuts off the Wnt/*β-*catenin signalling resulting in a decreased KIAA1199 transcript expression (Sabates-Bellver *et al*, 2007) as well as a downregulation of the ASCL2 transcript (Jubb *et al*, 2006). (2) Knockdown from ASCL2 results in the silencing of LGR5 (Van der Flier *et al*, 2009). Our data show that knockdown from KIAA1199 resulted in a decreased expression of (3) *β*-catenin, (4) ASCL2 and (5) LGR5 and to lesser extent some genes targeted by ASCL2 (not shown). A decreased *β*-catenin expression upon KIAA1199 knockdown may suggest a regulatory loop probably further affecting *β*-catenin signalling. (**C**) Immunofluorescence analysis confirmed the downregulation of *β*-catenin and ASCL2 upon KIAA1199 knockdown in SW480 cells compared with control cells (see also Supplementary Figure 4). Blue: DAPI nuclear stain; green: Alexa488 secondary antibody; × 630 magnification, Zeiss Axiovision, Carl Zeiss, Brock & Michelsen A/S, Birkerød, Denmark). (**D**) Western blot with extracts from SW480 cells stably transfected with an empty vector (lanes 2 and 4) or KIAA1199-sh3303 knockdown (lane 3, 5) was incubated with anti-*β*-catenin antibody in a 1 : 4000 (lane 2, 3) or 1 : 2000 dilution (lane 4, 5). *β*-catenin protein expression was markedly decreased upon KIAA1199 knockdown compared with control cells. *β*-actin was used as loading control. Lane 1=Marker Bio-Rad All Blue.

Lentiviral-mediated knockdown of KIAA1199 using five different shRNA constructs yielded between 45% and >80% knockdown efficiency in SW480 cells (Supplementary Figure 3a), whereas knockdown efficiency of SW948 and LS1034 cells with 70-fold higher endogenous KIAA1199 expression was only about 50% (data not shown). To investigate the transcriptional consequences associated with KIAA1199 knockdown, transcript profiling (U133plus2.0, ExonST.1.0arrays) of SW480 cells stably transfected with an empty vector or three different knockdown constructs with favourable knockdown efficiency sh6640 (>65%), sh2396 (>75%) and sh3303 (>80%) was performed to control for OFF-target effects (Supplementary Figure 3b). A total of 3727 probesets differentially expressed in common in all three knockdowns were identified (sh-3303 log_2_ ratio >∣0.5∣ accompanied by log_2_ ratio >∣0.2∣ in either sh6640 or sh2396 or both). The IPA analysis of this gene list revealed ‘cell to cell signalling’ and ‘cellular growth and proliferation’ were significantly impacted upon KIAA1199 knockdown (*P*<0.01). However, most interestingly, KIAA1199 knockdown affected the expression of 67 genes involved in Wnt/*β*-catenin signalling, most of them downregulated upon KIAA1199 knockdown (Figures 2a and b). This impact of KIAA1199 on Wnt-signalling was further substantiated by immunofluorescence microscopy (IF) and western blotting, showing a decreased protein expression of *β*-catenin and the Wnt-related stem cell marker ASCL2 upon KIAA1199 knockdown with construct KIAA1199-sh3303 (Figures 2c and d). Furthermore, IF confirmed the decrease of ASCL2 upon KIAA1199 knockdown when two additional, alternative knockdown constructs, sh2396 and sh6640, were used (Supplementary Figure 4a). A total of 145 genes were found to be systematically dysregulated in SW480 cells either upon KIAA1199 overexpression or knockdown with KIAA1199-sh3303 (log_2_>∣0.5∣, Supplementary Table 1). Hereof, 87 genes were increased upon KIAA1199 knockdown and decreased upon overexpression, among those the tumour suppressor p53. Another set of 58 genes were decreased upon knockdown and increased after KIAA1199 overexpression, among these several genes involved in Wnt-signalling including CD44, DKK3 or CST1.

### KIAA1199 knockdown impacts the cellular phenotype

**Figure 3:**
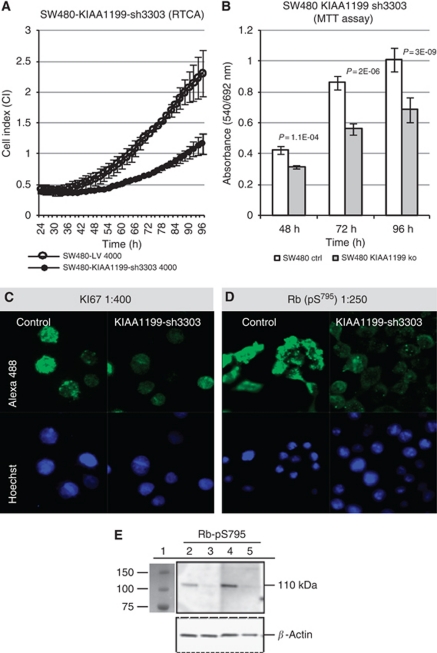
KIAA1199 knockdown affects proliferation of SW480 cells. SW480-KIAA1199-sh3303 colon cancer cells depleted of KIAA1199 (•) and an empty vector control (*○*) were seeded on E-plates on a RTCA-instrument (x-CELLigence, Roche). Experiments were performed at least in triplicates, values are shown as medians and s.d.'s for each group at selected times for a representative experiment. (**A**) Seeding of 4000 cells per well yielded control cells in the log phase from 36–96 h, cells were monitored in 1 min intervals. The proliferation rate of the KIAA1199-depleted cells was decreased by about 50% compared with control cells. (**B**) MTT-assays at selected time points confirmed a significant (*P*<0.05) decrease of proliferation of the KIAA1199-sh3303 depleted SW480 cells compared with control cells. (**C**) IF showed less nuclear expression of proliferation marker KI67 in KIAA1199-depleted SW480 cells compared with control cells using an 1 : 400 dilution of the anti-KI67 antibody (green: Alexa488-labelled secondary antibody; blue: Hoechst nuclear stain); × 630 (**D**) IF using a 1 : 250 dilution of the monoclonal anti-phosphoretinoblastoma antibody Rb-pS^795^ reacting specifically with Rb phosphorylated at serine 795 and not with non-phosphorylated Rb showed a dramatical decrease of phosphorylated Rb protein in KIAA1199-depleted SW480 cells compared with control cells. (**E**) Western blot with SW480 cells stably transfected with empty vector (lanes 2, 4) or KIAA1199-sh3303 knockdown (lane 3, 5), incubated with anti- RB-pS795 antibody (1 : 2000, lane 2, 3; 1 : 1000 lane 4, 5). Expression of the phosphorylated RB-pS795 protein was markedly decreased upon KIAA1199 knockdown compared with control cells. *β*-actin was used as loading control. Lane 1=Marker Bio-Rad All Blue.

Functional analyses using real time cell monitoring (RTCA) showed that knockdown using construct KIAA1199-sh3303 reduced the proliferation rate by about 50% (Figure 3a). The decrease in the proliferation rate was highly significant (*P*<0.0001) as confirmed by an MTT assay (Figure 3b), in agreement with reduced levels of proliferation-related transcripts. The typical granular nuclear staining of the proliferation marker KI67 (Figure 3c) as well as the strong nuclear expression of phosphoretinoblastoma (Figures 3d and e) were both decreased in KIAA1199-sh3303-depleted SW480 cells. Moreover, KIAA1199 knockdown using alternative constructs sh6640 and sh2396 confirmed these results. IF showed a markedly decreased KI67 and Rb-pS795 in KIAA1199 protein expression in KIAA1199-depleted SW480 cells although an MTT assay confirmed a significantly (*P*<0.05) reduced proliferation rate (Supplementary Figures 4a and b).

Moreover, pathway analysis pointed out that KIAA1199-sh3303 knockdown reduced the actin filament or microtubule-based cellular movement comprising genes like *MYO1E*, *KIF1B* or *TUBE1* as well as the maintenance of the cytoskeleton signalling involving genes like *MTSS1*, *ARHGAP26*, *DIAPH2*, *ABLIM1* or *DAAM1*). RTCA-migration analysis of KIAA1199-depleted SW480 cells using CIM-plates showed a decreased migration (Supplementary Figure 5a). Moreover, IPA analysis showed that molecules modulating cell–cell contacts were altered upon KIAA1199 knockdown. RTCA analysis showed a 50% reduced cellular adhesion of KIAA1199-depleted SW480 cells (Supplementary Figure 5b). Decreased adhesion was confirmed by a colorimetric assay on fibronectin-coated plates (Supplementary Figure 5c).

### *In vitro* impact of KIAA1199 on Wnt-signalling molecules is reflected in the clinical CRC samples

*In vitro* data suggested the existence of a potential correlation between the expression of KIAA1199 and Wnt-signalling molecules. Transcript data from a set of nine normal mucosas and 18 microdissected MSS-adenocarcinomas analysed on Exon1.0ST-arrays (set 4, (Thorsen *et al*, 2008)) showed that KIAA1199 transcript expression correlated very well with the expression of 37 genes known to be involved in Wnt/*β*-catenin-signalling (Pearson's coefficient 0.5–0.9) (Table 1). The KIAA1199 transcript expression correlated best with Cyclin D1 (CCND1), LGR5, AXIN2, ASCL2, CD44, PITX2 and CLDN1 ((Pearson's coefficient >0.8), Supplementary Figure 6a). Remarkably, these genes were also found to be deregulated upon KIAA1199 knockdown (Table 1). Excluding the normal samples, KIAA1199 expression still correlated well with the Wnt-signalling genes *CD44*, *LGR5* and *β-catenin*, supporting that KIAA1199 has a strong relation to the Wnt/*β-*catenin signalling pathway.

We compared nuclear KIAA1199 protein expression with nuclear *β*-catenin expression in the same cores of our CRC TMA, as nuclear *β*-catenin corresponds to active Wnt/*β-*catenin signalling *in vivo.* Remarkably, 69% (25 out of 36) of the tumours with strong nuclear and weak cytoplasmic KIAA1199 localization showed nuclear *β*-catenin localization, compared with 51% (106 out of 209) of the tumours with cytoplasmic KIAA1199 expression only (Supplementary Figure 6b). The nuclear KIAA1199 protein location was significantly correlated with the presence of nuclear *β*-catenin (odds ratio, 2.13; *P*=0.014) in logistic regression analysis.

## Discussion

The KIAA1199 transcript was strongly upregulated in 95% of the colon adenocarcinomas compared with normal mucosas in a total of 511 colon samples investigated. Array-based methylation analysis and comparison with transcript expression data provided evidence that KIAA1199 expression may have been shut down in normal colon mucosa by promotor methylation.

In colon cancer, we localised strong expression of the KIAA1199 protein in the cytoplasm and the nucleus of adenocarcinomas. This is different compared with gastric cancer, where the protein was exclusively localised in the cytoplasm (Matsuzaki *et al*, 2009). Moreover, in gastric cancer the overall 5-year survival rate was significantly better for patients with low KIAA1199 expression in the primary tumour and strong KIAA1199 expression was more frequent in patients with distant metastasis and peritoneal dissemination (Matsuzaki *et al*, 2009). On the contrary, in our study on colon cancer, strong KIAA1199 expression was generally not significantly correlated to lower overall survival or the development of liver or lung metastases in patients with stage II primary tumours (patients developing peritoneal dissemination were excluded from our analyses). However, strong nuclear expression favoured disease-free survival in a subgroup of the stage II patients suggesting a role for nuclear KIAA1199 in CRC.

The finding of better survival in a minor group with high KIAA1199 nuclear expression may point to the diversity in biological pathways being active during carcinogenesis and during the metastatic process. There is no doubt that Wnt-signalling is important during CRC cancer formation. However, during the metastatic process, the generally abnormal genome, as well as the complex pathway networks, may show unexpected findings. In this case, the proliferative and mobility aspects of KIAA1199 seem to be uncoupled from the metastatic process. Recent findings provide evidence for the existence of alternative pathways counteracting Wnt/*β*-catenin signalling. For example, Lee *et al*, (2010) show that the glycoprotein 90K has antitumour activity in CRC cells suppressing Wnt/*β-*catenin signalling by ISGylation-dependent ubiquitination of *β*-catenin when interacting with CD9/CD82.

Nuclear KIAA1199 correlated significantly with the expression of nuclear *β*-catenin in colon adenocarcinomas. However, KIAA1199 lacks a nuclear import signal as well as predicted DNA-binding properties. Due to a transmembrane region, KIAA1199 may bind to other KIAA proteins containing domains localised to highly specialized submembranous sites, indicating a participation in intracellular signalling events (Nakayama *et al*, 2002). KIAA1199 interacts, for example, with KIAA0463, a plexin 2 precursor, which is a co-receptor for semaphorin involved in remodelling of the cytoskeleton, invasive growth and cell migration (Zachary *et al*, 2009). The KIAA1199 protein precursor is phosphorylated by the anaplastic lymphoma kinase (Daub *et al*, 2008), suggesting a potential role for KIAA1199 in phosphorylation networks and intracellular signalling processes.

Previously, KIAA1199 expression in colon adenomas was correlated to the expression of 21 known Wnt-signalling genes (Sabates-Bellver *et al*, 2007). In our study, KIAA1199 transcript expression correlated with the expression of 37 genes known to be involved in Wnt-signalling, strongly supporting an involvement of KIAA1199 in the Wnt/*β*-catenin pathway.

We showed that KIAA1199 overexpression in SW480 cells affected expression of genes involved in the Wnt/*β*-catenin pathway. About 25% of the genes differentially expressed upon KIAA1199 overexpression were also significantly differentially expressed in adenocarcinomas compared with normal mucosas, indicating a potential regulatory role for KIAA1199 *in vivo*. However, KIAA1199 overexpression did not have a major impact on the cells as it would have been expected from an 80-fold upregulation. One explanation may be that in SW480 cells an increase in KIAA11199 probably only results in minor changes due to a constitutively activated Wnt/*β*-catenin signalling.

The number of genes involved in cell-cycle regulation differentially expressed upon KIAA1199 knockdown, decreased proliferation and the decrease of KI67 and RbpS795 suggested that KIAA1199 knockdown may affect cell-cycle progression. This may potentially be achieved by affecting the EBP1–RB-E2F transcription repression as unphosphorylated RB will inhibit the activity of the E2F family of transcription factors (Sidle *et al*, 1996) and will thus lead to a decelerated G1-S transition (Lee and Yang, 2003).

Alternatively, ASCL2, identified to be downregulated upon KIAA1199 knockdown, may promote progression through the G2/M cell-cycle checkpoint and may be a putative regulator of proliferation overexpressed in intestinal neoplasia (Jubb *et al*, 2006). ASCL2 is located in stem cells and its knockdown results in the loss of stem cells in the mouse intestine of an *in vivo* model (Van der Flier *et al*, 2009). Both, ASCL2 and KIAA1199 are under *β*-catenin control (Sabates-Bellver *et al*, 2007). Our data suggest that KIAA1199 may affect the expression of ASCL2 and LGR5 and may thus be an important player in functional Wnt/*β*-catenin signalling. In addition, KIAA1199 knockdown resulted in a feedback-loop of negative regulation of *β*-catenin in SW480 cells. The attenuation of the Wnt/*β*-catenin signalling may also contribute to a decrease in proliferation. Interestingly, despite the constitutive activation of Wnt/*β*-catenin signalling in SW480 cells, the knockdown of the KIAA1199 transcript was sufficient to cause an impact on the Wnt/*β*-catenin pathway and, as a consequence, the observed cellular phenotype. This suggests a central role for KIAA1199 in Wnt/*β*-catenin signalling.

The TGF-*β* and Wnt-signalling pathways can independently or cooperatively regulate LEF1/TCF target genes (Letamendia *et al*, 2001). Recent studies reported that SMAD4/DPC4 has a TGF-*β*-independent function as tumour suppressor in cooperation with *β*-catenin/Lef to regulate target gene expression (Tian *et al*, 2009). *In silico* promotor analyses of the KIAA1199 promoter region identified binding sites, for example, TP53, LEF1/TCF or SMAD4 (Supplementary Table 2). Reconstitution of functional SMAD4 protein in SW480 cells (Stuhler *et al*, 2006) resulted in a 10-fold upregulation of the KIAA1199 transcript, expression was confirmed by IF and western blot (not shown), suggesting an involvement of the SMAD4/TGF-*β* signalling pathway as an alternative regulatory mechanism for KIAA1199 expression *in vivo* in the presence of functional SMAD4. Alternative regulatory mechanisms for KIAA1199 may exist, for example, the COX2-signalling cascade. Treatment of HT29 cells with a selective cyclooxygenase-2 (COX2) inhibitor resulted in a decrease of the KIAA1199 transcript level and an anti-proliferative effect (Galamb *et al*, 2010).

In conclusion, we provide evidence that KIAA1199 transcript and protein are highly expressed in the majority of CRCs. KIAA1199 probably participates in Wnt-signalling, affecting cell proliferation, motility and adhesion. Moreover, KIAA1199 has a clinical correlation to outcome in stage II CRC patients. These findings warrant further studies to understand KIAA1199's direct molecular interactions as well as to investigate whether KIAA1199 may be a potential biomarker or therapeutic target.

## Figures and Tables

**Table 1:** Genes involved in Wnt/*β*-catenin signalling correlated with the expression of KIAA1199 in normal mucosa and adenocarcinomas (Pearson's correlation coefficient ⩾0.5) analysed on different 37 genes for Exon 1.0 ST array, 32 genes for U133plus2.0 array.

**Pearson's correlation coefficient**					
**Exon 1.0 ST array^a^**	**U133plus2.0^b^**	**Symbol**	**KIAA1199 targeted**	**Genbank**	**Gene description**
0.9	0.8	CLDN1		AF101051	Claudin 1
0.8	0.9	CD44	^*^	BC004372	CD44
0.8	0.7	MYC	^*^	BC000141	v-myc myelocytomatosis viral oncogene homologue (avian)
0.8	0.7	ETV4		BC016623	Ets variant gene 4 (E1A enhancer-binding protein, E1AF)
0.8	0.8	AXIN2	^*^	BC101533	Axin 2 (conductin, axil)
0.8	0.8	ASCL2	^*^	BC057801	Achaete-scute complex homologue 2 (Drosophila)
0.8	0.8	CCND1	^*^	BC023620	Cyclin D1
0.8	0.7	EPHB3		BC052968	EPH receptor B3
0.7	0.7	HIG2		AF144755	Hypoxia-inducible protein 2
0.7	0.7	NKD1	^*^	BC051288	Naked cuticle homologue 1 (Drosophila)
0.7	0.7	SOX9	^*^	BC056420	SRY (sex-determining region Y)-box 9
0.7	0.8	PITX2		AK127829	Paired-like homeodomain 2
0.7	0.7	AXIN1		AB208876	Axin 1
0.7	0.7	CSNK1E	^*^	BC006490	Casein kinase 1, epsilon
0.7	0.6	EPHB2	^*^	AF025304	EPH receptor B2
0.7	0.6	LGR5	^*^	AK075399	Leucine-rich repeat-containing G protein-coupled receptor 5
0.7	0.6	STRA6	^*^	BX537413	Stimulated by retinoic acid gene 6 homologue (mouse)
0.6	0.6	IL8		M17017	Interleukin 8
0.6	0.6	TCF7		AL834166	Transcription factor 7 (T-cell specific, HMG-box)
0.6	0.6	BIRC5	^*^	BC034148	Baculoviral IAP repeat-containing 5 (survivin)
0.6	0.5	BCL9		BC116451	B-cell CLL/lymphoma 9
0.6	0.7	MMP7		BC003635	Matrix metallopeptidase 7 (matrilysin, uterine)
0.6	0.7	MSX2∣MSX2P1		D89377	msh homeobox 2 ∣ msh homeobox 2 pseudogene 1
0.6	0.6	WISP1		AF100779	WNT1 inducible signalling pathway protein 1
0.6	0.6	MET	^*^	BC130420	Met proto-oncogene (hepatocyte growth factor receptor)
0.6	0.6	SOX4		BC072668	SRY (sex-determining region Y)-box 4
0.6	<0.5	DVL3		U75651	Dishevelled, dsh homologue 3 (Drosophila)
0.6	<0.5	APCDD1	^*^	BC053324	Adenomatosis polyposis coli down-regulated 1
0.5	0.6	FZD3	^*^	AB039723	Frizzled homologue 3 (Drosophila)
0.5	<0.5	PTTG1∣PTTG2		AF095288	Pituitary tumor-transforming 1 ∣ 2
0.5	0.5	WNT2		BC078170	Wingless-type MMTV integration site family member 2
0.5	0.6	RARG		AK290588	Retinoic acid receptor, gamma
0.5	0.5	WNT5A	^*^	BC064694	Wingless-type MMTV integration site family, member 5A
0.5	0.5	EDAR	^*^	AF130988	Ectodysplasin A receptor
0.5	<0.5	FZD6		BC060836	Frizzled homologue 6 (Drosophila)
0.5	<0.5	MMP3		AK223291	Matrix metallopeptidase 3 (stromelysin 1)
0.5	0.5	LRP5	^*^	AF077820	Low-density lipoprotein receptor-related protein 5

Abbreviations: CLL=chronic lymphocytic leukemia; MSS=microsatellite stable.

Heroff, 18 genes marked with an asterisk were differentially expressed upon KIAA1199 knockdown in SW480 cells. (^*^log_2_ ratio ∣>0.5∣, *P*<0.05).

a mRNA from nine normal mucosas and 18 microdissected MSS adenocarcinomas.

b mRNA from 10 normal mucosas and 301 MSS adenocarcinomas.
