# Supplementary figures and images for: Repression of KIAA1199 attenuates Wnt-signalling and decreases the proliferation of colon cancer cells
Source: Br J Cancer. 2011 Jul 19;105(4):552–61. doi: 10.1038/bjc.2011.268 (PMC3170968; doi:10.1038/bjc.2011.268)

Supplementary Figure 1

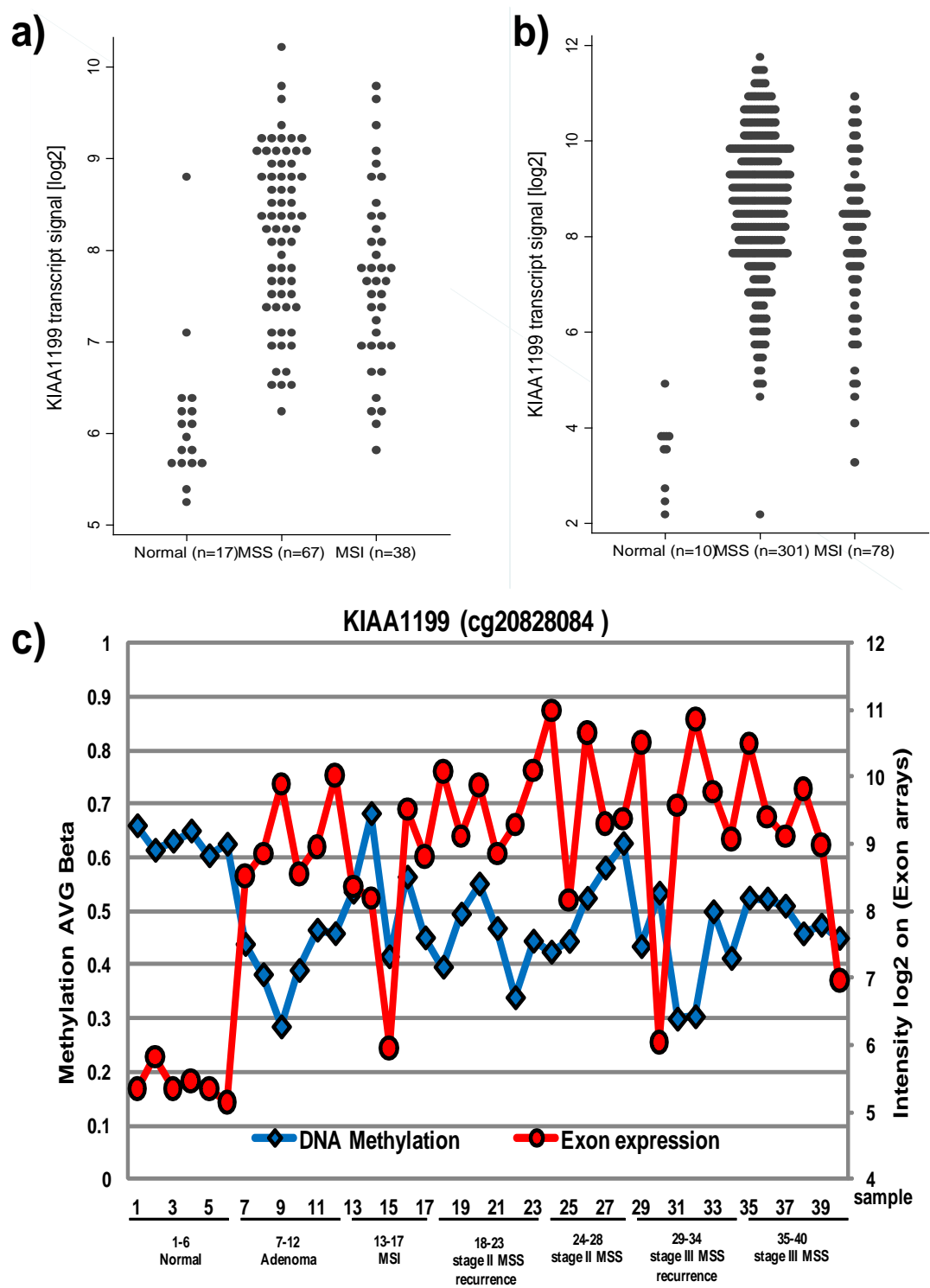

Supplement: Supplementary Figure 1 [file bjc2011268x1.pdf]

## Supplementary Figure 2

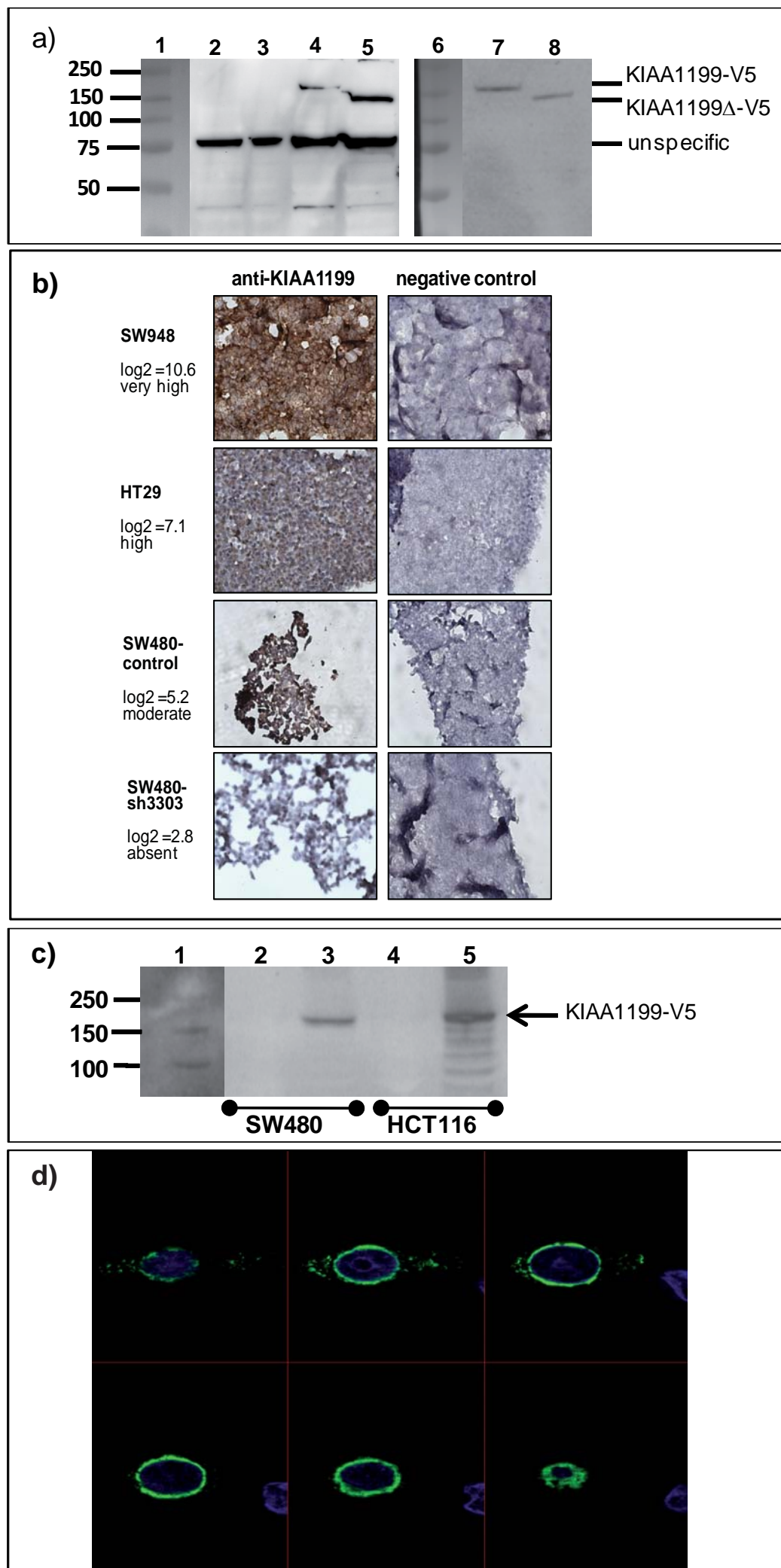

Supplement: Supplementary Figure 2 [file bjc2011268x2.pdf]

## Supplementary Figure 3

### a) Endogenous KIAA1199 expression

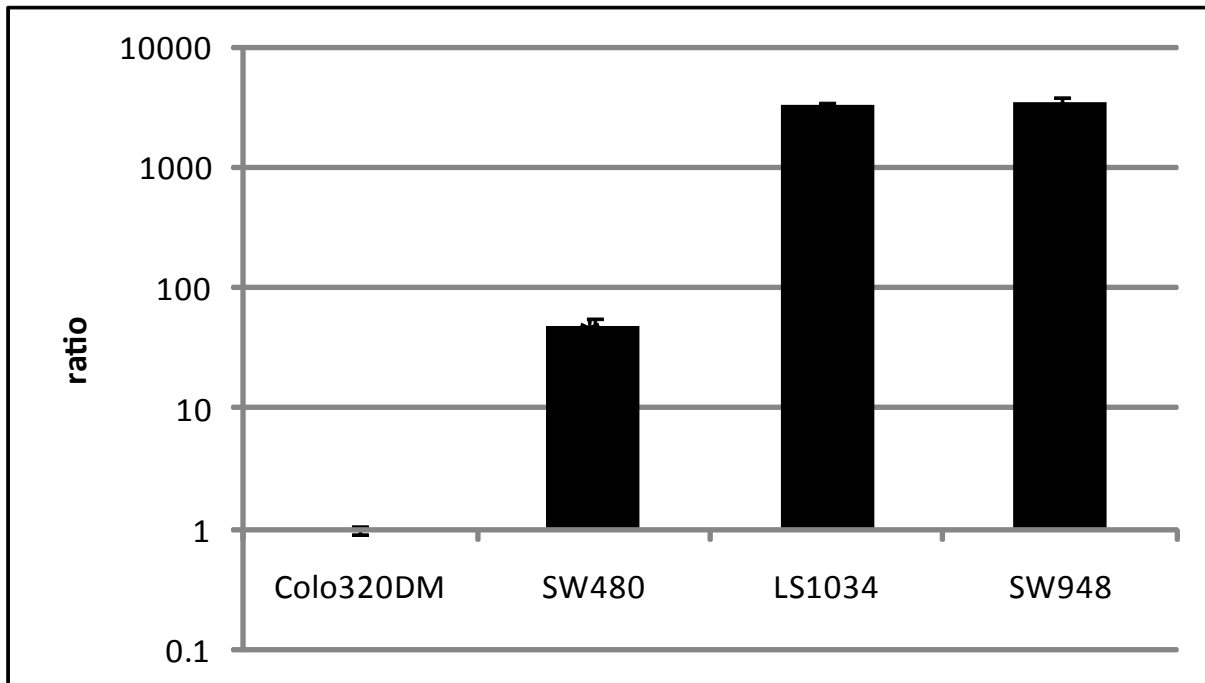

### b) Stable KIAA1199 knockdown in SW480 cells

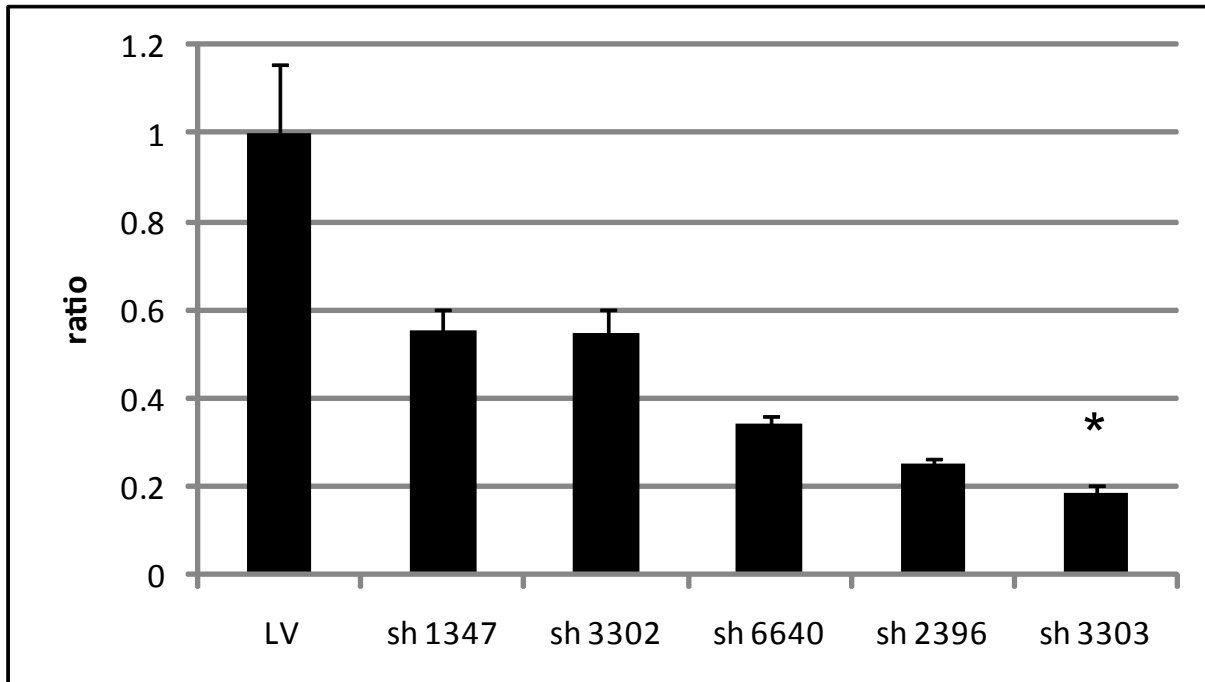

Supplement: Supplementary Figure 3 [file bjc2011268x3.pdf]

Supplementary Figure 4

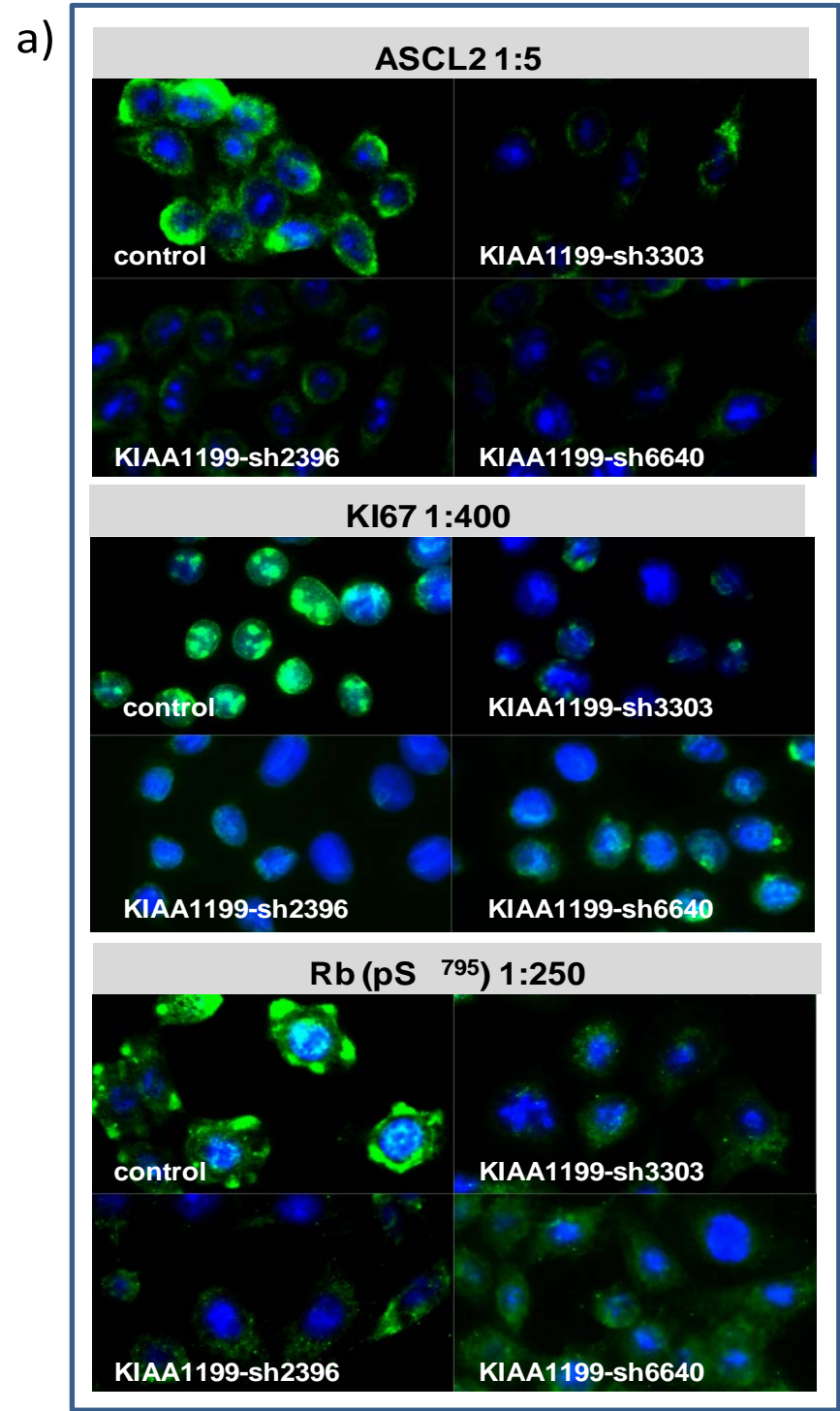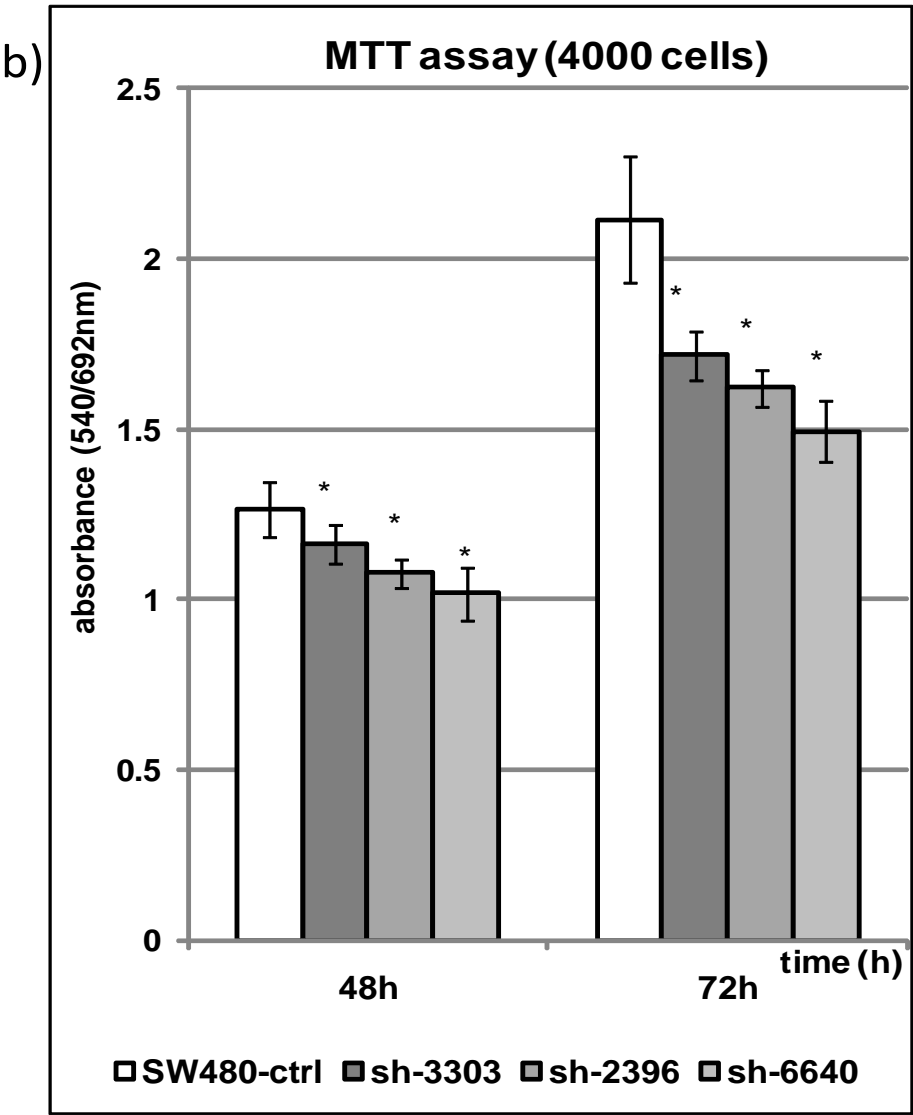

Supplement: Supplementary Figure 4 [file bjc2011268x4.pdf]

# Supplementary Figure 5

a) Cell migration

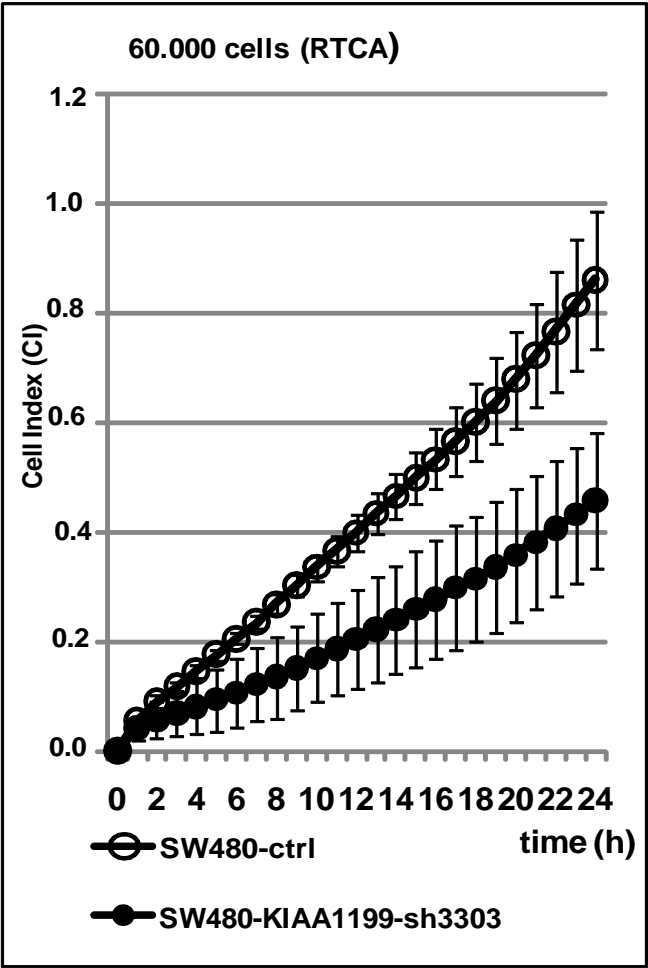

b) Cell adhesion

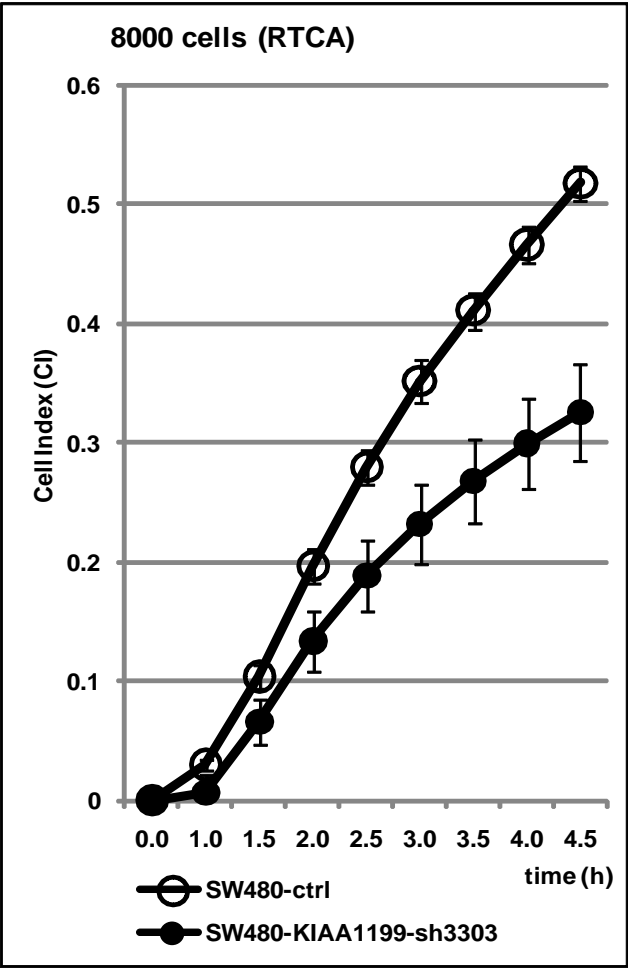

c) Cell adhesion

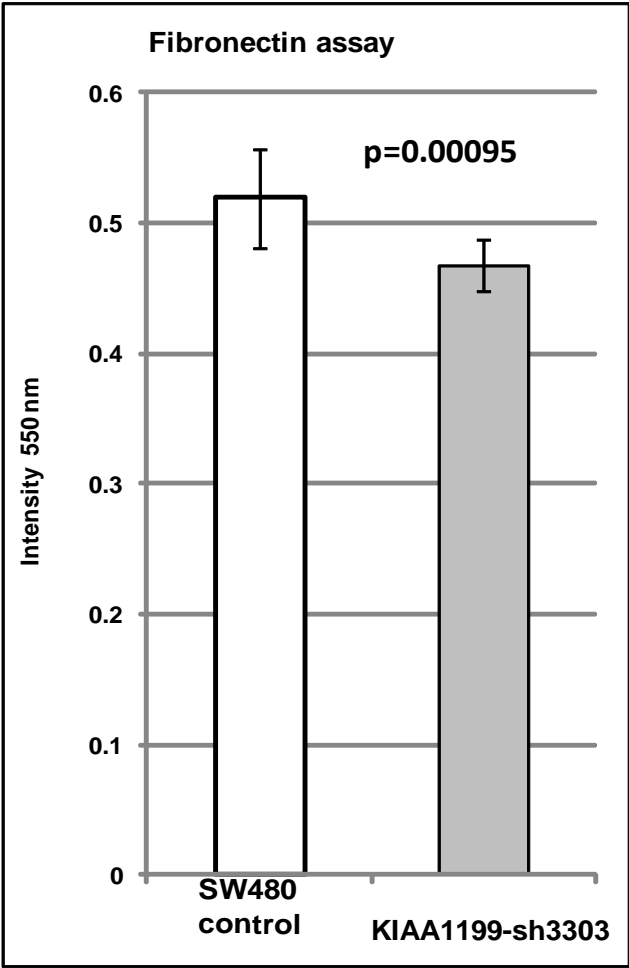

Supplement: Supplementary Figure 5 [file bjc2011268x5.pdf]

Supplementary Figure 6

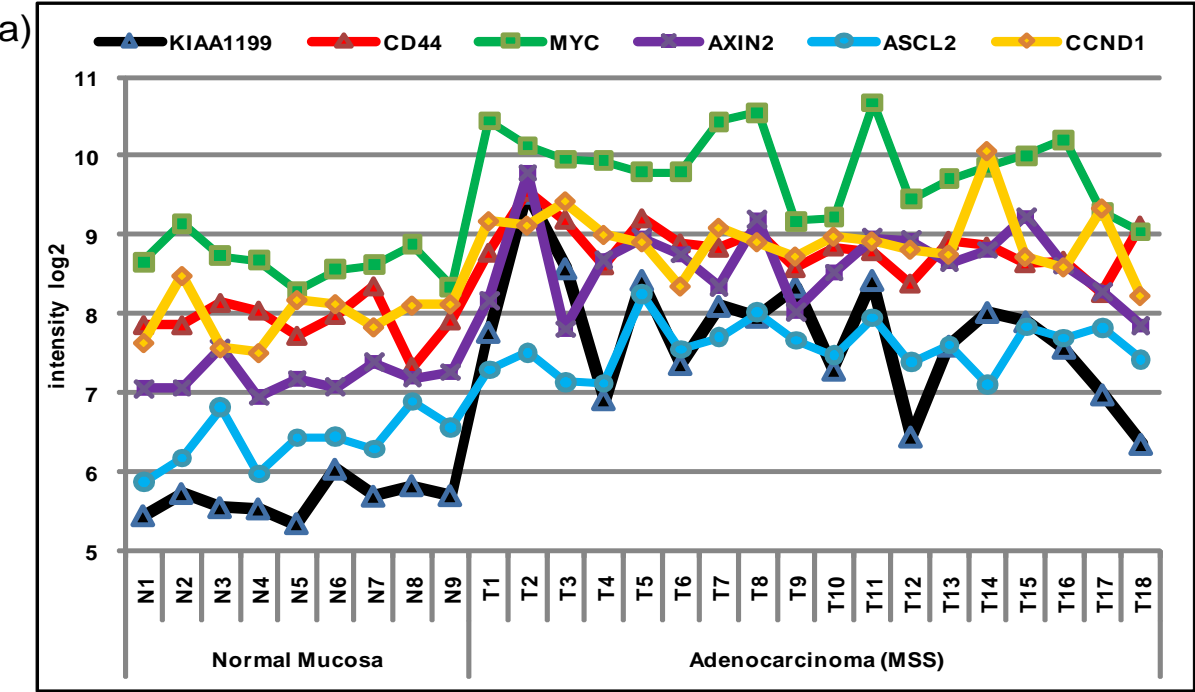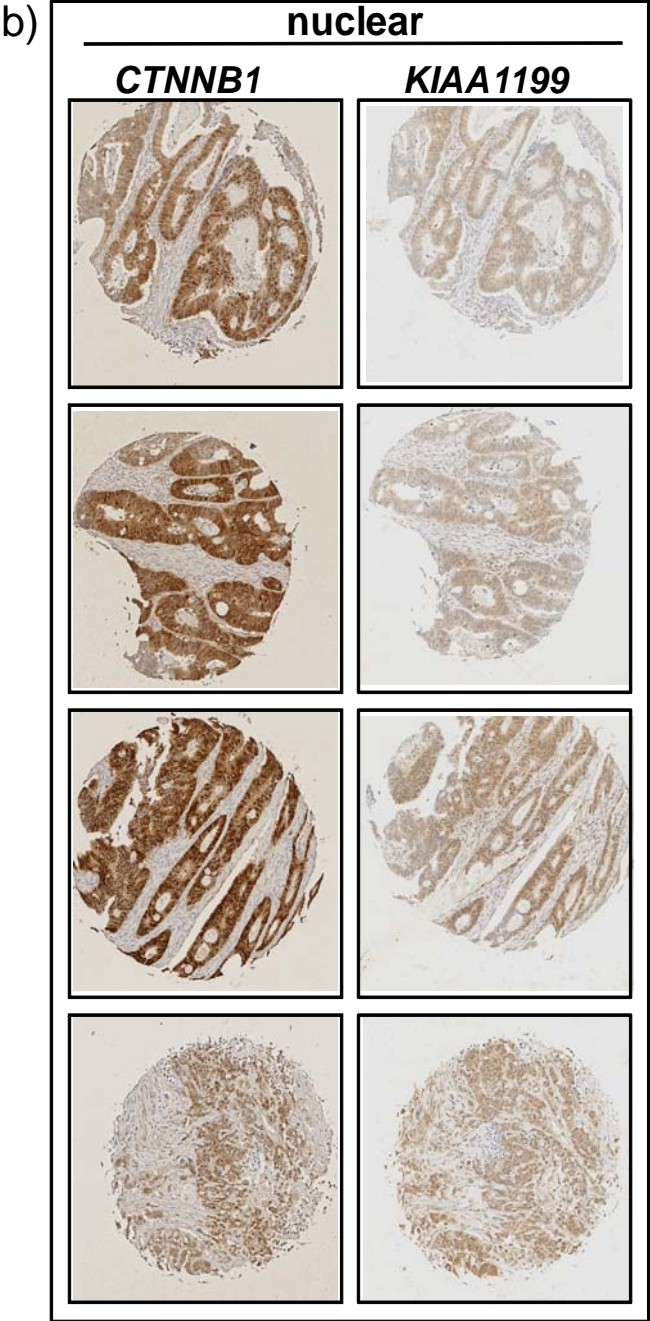

Supplement: Supplementary Figure 6 [file bjc2011268x6.pdf]
