# Supplementary material for: Repression of KIAA1199 attenuates Wnt-signalling and decreases the proliferation of colon cancer cells
Source: Br J Cancer. 2011 Jul 19;105(4):552–61. doi: 10.1038/bjc.2011.268 (PMC3170968; doi:10.1038/bjc.2011.268)
Supplement: Supplementary Data [file bjc2011268x9.pdf]

MD20113120 – British Journal of Cancer

Repression of KIAA1199 attenuates Wnt-signalling and decreases the proliferation of colon cancer cells

Birkenkamp-Demtroder et al

SUPPLEMENTARY DATA

Cloning of KIAA1199 and characterisation of antibody specificity. Cloning of the full length human KIAA1199 transcript as a V5-HIS tagged protein identified a novel splice isoform △KIAA1199 with 28 exons, skipping exon 25, resulting in a TGA stop codon at position 3898 due to a frame shift. The resultant peptide sequence "KQRTISWR" at position G1136 constitutes a truncated protein of 1144 amino acids lacking the C-terminal GG domain. This splice variant with unknown function was detected in three out of twenty human primary colon adenocarcinomas analyzed (data not shown). The affinity-purified, monospecific anti-KIAA1199 antibody was specific for the full length and the truncated KIAA1199 variant (supplementary Figure 2a). As no clinical correlation was found for the splice variant, all studies were restricted to full length KIAA1199 only.

KIAA1199 expression and localization in colon cell lines. Microarray profiling identified no KIAA1199 expression in HCT116, HCT15 and CaCo2 cells, moderate expression in SW480 cells and high expression in HT29, Colo205 and SW620 cells (data not shown), thus the KIAA1199:V5-HIS protein was transiently overexpressed in HCT116 and SW480 cells (supplementary Figure 2b). Immunofluorescence microscopy and z-stacking of SW480 cells overexpressing KIAA1199:V5-HIS showed a protein accumulation in the cytoplasm and in close proximity to the nuclear envelope

(supplementary Figure 2c). Moreover, we found that KIAA1199 overexpressing SW480 cells secreted the protein into the culture media as identified on Western blots of cell culture supernatants (not shown). Analysis of human plasma samples derived from CRC patients depleted from IgG and albumin using the anti KIAA1199 antibody unfortunately failed to identify the protein in the plasma by Western blotting (not shown). However, the KIAA1199 synonym CCSP1 (colon cancer secreted protein 1) had been proposed to be a novel serological marker of colon neoplasia (Fink *et al.*, 2006) and recently, the KIAA11199 protein had been identified in exosomes (http://exocarta.ludwig.edu.au/) (Mathivanan and Simpson, 2009), suggesting a potential secretion of KIAA1199 into the plasma of CRC patients via small membrane vesicles.

SUPPLEMENTARY FIGURES

Supplementary Figure 1. Sample distribution of KIAA1199 transcript profiling data and methylation analysis

a) 122 samples analyzed on U133A GeneChips; Normal mucosa (n=17) [95% CI 5.71-6.54]; MSS (n=67) [95% CI 7.97-8.42]; MSI (n= 38) [95% CI 7.37-8.03];

b) 389 samples analyzed on U133plus2 GeneChips; Normal mucosa (n=10) [95% CI 2.91-4.08], MSS (n=301) [95% CI 8.51-8.86], MSI (n= 78) [95% CI 7.64-8.36];

c) Genome-wide transcript profiling using Exon1.0ST-arrays in combination with DNA Methylation profiling using Infinium HumanMethylation27 BeadChips (described in detail in (Øster B *et al.*, 2011)) of an additional set of 40 microdissected colon tissue samples (normal, adenoma, and adenocarcinoma) revealed an inverse correlation between KIAA1199 promotor methylation and expression level (r= -0.542, Pearson).

Original Supplementary Figure 2 was removed. Original Supp. Fig 3 was replaced by this supp Fig 2 etc.

Supplementary Figure 2 Characterisation of antibody specificity and KIAA1199 protein expression *in vitro*.

a) Cloning of the KIAA1199 gene identified a full length KIAA1199 and truncated △KIAA1199 transcript variant. Both KIAA1199 variants were overexpressed in colon cancer cell lines. Western blots with extracts from SW480 cells transiently transfected with a control with empty vector (lane 2, 3), with a vector encoding the KIAA1199-V5-HIS full length protein (lane 4, 7) or the truncated KIAA1199△-V5-HIS splicevariant (lane 5, 8). Lane 1= Marker "All Blue" (BioRad, Denmark).

The unpurified rabbit-polyclonal antiserum detected the KIAA1199-V5 full-length protein (157kDa upper band), and the KIAA1199△-V5 splicevariant with a lower molecular weight. The unpurified antiserum also detected an unspecific band of about 80 kDa in the untransfected as well as the KIAA1199 transfected cells.

The monospecific antibody, affinity purified from rabbit-polyclonal antiserum was highly specific for both, the KIAA1199 full-length protein (upper band, 157kDa), and the △KIAA1199 splicevariant (133kDa), no unspecific bands were detected.

b) The antibody specificity of the monospecific antibody was proven by Immunohistochemistry using a panel of Formalin fixed paraffin embedded (FFPE) colon cell lines with different KIAA1199 transcript expression levels (given as log2 intensities) as previously assessed by microarray expression profiling on Affymetrix arrays. KIAA1199 localized in the nucleus and the cytoplasm of SW948, HT29 and SW480-ctrl colon cancer cells, KIAA1199 was not detected in the SW480-sh3303 KIAA1199 knockdown cells (magnification 400x).

c) Western blot of extracts from SW480 (lane 2,3) or HCT116 cells (lane 4,5), transiently transfected with an empty vector as control (lane 2,4) or with a vector encoding the full-length KIAA1199-V5-HIS protein (lane 3, 5). The KIAA1199 protein was detected using an anti-V5 antibody. Lane1= Marker “All Blue” (BioRad, Denmark).

d) Immunofluorescence microscopy of SW480 cells overexpressing full-length KIAA1199-V5-His. Images were acquired with an Axiovert 200M (630x) and ApoTome Slider and Axiovision Rel. 4.8 (Zeiss, Birkerød, Denmark). Z-stacking (1 µm) identified KIAA1199 in the cytoplasm (green, Alexa488 secondary antibody) and revealed an accumulation of KIAA1199 in the nuclear membrane and/or in close proximity to the nuclear envelope. Blue: DAPI nuclear stain;

Supplementary Figure 3 KIAA1199 expression in colon cell lines. a) Four different cell lines were analyzed by QRT-PCR for KIAA1199 expression. LS1034 and SW948 showed extreme high KIAA1199 expression levels, while SW480 showed moderate expression levels. b) Lentiviral mediated knockdown of KIAA1199 was achieved by five different shRNA constructs. Construct sh-3303 resulted in an >80% knockdown of the KIAA1199 transcript.

Supplementary Figure 4. Impact of KIAA1199 knockdown on proliferation and protein expression using three knockdown constructs. To exclude OFF-target effects and to ensure that the identified expression changes and effects on the phenotype are specific, all experiments were performed with three different KIAA1199 knockdown constructs with different knockdown efficiency: sh6640 (>65%), sh2396 (>75%) and sh3303 (>80%).

a) Immunofluorescence analysis of control cells and three different KIAA1199 knockdowns (sh3303, sh2396 or sh6640) confirmed that the downregulation of ASCL2 was specifically induced by KIAA1199 knockdown. Moreover, all three KIAA1199 knockdowns induced less nuclear expression of the proliferation marker KI67 and the

phosphorylated form of the cell cycle protein RBpS795 compared to SW480 control cells; blue: DAPI nuclear stain; green: Alexa488 secondary antibody; 630x magnification, Zeiss Axiovision)**.**

b) SW480 with stable constructs of either a control or three knockdowns (sh3303, sh2396 or sh6640) were seeded on a 96-well microtiter plate with 4000 cells/well. MTT-assays at 48h and 72h post transfection showed a significant ($p<0.05$, marked with asterisks) decrease of proliferation with all three knockdowns compared to control cells with an empty vector (two-sided t-test KIAA1199-**sh3303** 48h p=0.006, 72h p=3.2E-05; KIAA1199-**sh2396** 48h p=7.9E-06, 72h p=4.4E-06; KIAA1199-**sh6640** 48h p=4.9E-07, 72h p=1.3E-07).

Supplementary Figure 5. KIAA1199 depletion affects cellular adhesion and migration of SW480 cells. a) For migration studies, 60.000 SW480 control or KIAA1199-sh3303 cells per well were seeded on CIM plates with and without 10% FCS in the lower chamber and migration was monitored in RealTime over a time period of 24h. KIAA1199-sh3303 depletion decreased migration of SW480 cells compared to cells with an empty vector control. b) KIAA1199-sh3303 knockdown decreased adhesion within the initial 4 hours after seeding (8000 cells per well seeded). The differences in adhesion disappeared within 24 h post-seeding. c) A colorimetric cell adhesion assay using Fibronectin coated plates showed a minor, but highly significant decrease of adhesion in KIAA1199-sh3303 depleted cells.

Supplementary Figure 6 KIAA1199 correlation with Wnt/beta-catenin signalling genes. a) 27 microdissected samples were expression profiled on Exon1.0ST-arrays; Normal mucosa (n=9) and MSS adenocarcinomas (n=18). *KIAA1199* expression was correlated to the expression of genes involved in Wnt/beta-catenin signalling. Highest correlation was identified for *ASCL2, CD44, MYC, AXIN2* and *CCD1* (Pearson coefficient > 0.8). These genes were also

targeted upon KIAA1199 knockdown (Table 1). b) Beta-catenin expression was analyzed on our colon TMA and its nuclear expression was correlated with nuclear KIAA1199 expression. Nuclear KIAA1199 protein location showed a significant correlation with the presence of nuclear beta-catenin (odds ratio, 2.13; p=0.014) in logistic regression analysis. Examples of four adenocarcinomas with nuclear localization of beta-catenin (CTNNB1) accompanied by nuclear localization of KIAA1199 in the same tissue sample are shown.

SUPPLEMENTARY TABLE LEGENDS

Supplementary Table 1 145 genes inversely regulated in SW480 cells upon KIAA1199 knockdown or overexpression, respectively above a treshold of log2 > |0.5| in at least one of the samples, either knockdown or overexpression.

Supplementary Table 2 Identification of potential binding factors potentially regulating KIAA1199 expression. In silico promotor analyses of the (+) strand sequence of the KIAA1199 promoter region using Genomatixx software applying a threshold score of >0.9 (Cartharius *et al.*, 2005) identified 20 significant hits +/- 250 nt around the TATA sequence, comprising target sequences for e.g. KLF, TCF12 and MYC. Moreover, 77 significant hits were identified in the region 1500nt upstream, comprising binding sites for e.g. p53, klf, lef1/tcf, smad3 or smad4.

SUPPLEMENTARY METHODS

Blast of Probe set sequences. Sequences of the KIAA1199 probe sets on all Affymetrix GeneChips were BLASTed against the human KIAA1199 sequence NM_018689.1. Hu35KsubC: RC_N71781_at, position 2017-2310 coding region; HG-U133A: 212942_s_at, position 6549-6996 3’UTR, 239746_at and 239747_s_at

position Chr 15 (not KIAA1199); HG-U133_PLUS_2: 1554685_a_at position 2992-3523 in coding region, 212942_s_at position 6549-6996 3'UTR, 239746_at and 239747_s_at position Chr 15 (not KIAA1199). The probe sets used for analysis were located in the coding region or the 3'UTR region of the KIAA1199 transcript.

Prediction of the KIAA1199 function. SignalP 3.0 prediction software (DTU) identified an N-terminal signal peptide at position 1-30 with the most likely cleavage site between aa 30 and 31 (probability: 0.993). KIAA1199 contains four PbH1-repeats and seven predicted N-glycosylation sites (NetNGlyc1.0, DTU), 23 predicted phosphorylation sites (threshold >0.9, NetPhos2.0, DTU) and six predicted protein-kinase-C (PKC) specific phosphorylation sites (threshold >0.8), the highest score of 0.92 at residue T-891.

Cloning and overexpression of human KIAA1199 and △KIAA1199. A full-length cDNA sequence was retrieved by BLASTing the target sequence RC_N71781_at from the Hu35KsubC-array. Primers (forward 5'-GCCACGATGGGAGCTGCTGGGAGG and reverse CAACTTCTTCTTCTTCACCACAGGGAT) contained the start codon and last amino acid 1361_leu of the human hKIAA1199 gene (NM_018689.1) and were used to clone the coding cDNA in frame with a V5-His C-terminal tag or a GFP tag. The PCR products were inserted into pcDNA 3.1 bidirectional and pcDNA3.1/V5-His-TOPO vectors, respectively, using Eukaryotic TA Expression Kits (Invitrogen Corp., Carlsbad, CA). Transient transfection with pcDNA 3.1/ V5-His:KIAA1199 or pcDNA 3.1/ GFP:KIAA1199 of human colon cancer cell lines HCT116 and SW480 was achieved using Lipofectamin (Invitrogen), following the manufacturer's instructions. In addition to the full length transcript, a splicing isoform △KIAA1199 was identified. For validation, cDNA from five MSS and five MSI adenocarcinoma samples as well as their matching normal mucosas (Mansilla *et al.*, 2007) were synthesized and the KIAA1199 fragment expanding exons 16 to 25 was amplified (primers forward

CACGTACCAACGGGCCCCTC, reverse GTCCTTTGTTTTCGTGAGTCCT). The reverse primer bridges exon 24 and 26 and is specific for the truncated isoform.

shRNA-vector preparation, lentivirus production and lentiviral infection. The shRNA vector constructs were produced with pLKO.1 puro (kindly provided by Sheila Stewart) containing a 1.8 kb stuffer sequence in place of the shRNA cassette (Stewart *et al.*, 2003). The pLKO.1 plasmid was doubly digested by EcoRI and AgeI for 1 hours to release the 1.8 kb stuffer fragment. DNA fragments were then separated using a 1% agarose gel. The 7.1 kb EcoRI/AgeI band was excised and DNA was extracted using Qiaquick gel extraction kit (Qiagen). Pairs of sense and antisense hairpin oligonucleotides were obtained from Eurofins MWG Operon. To form the shRNA cassette 5.4 µg of each oligonucleotide was annealed in a volume of 100 µl. The annealing buffer was 1x T4-DNA ligase buffer (Invitrogen, Karlsruhe, Germany). The annealing mixture was incubated in a thermal cycler (DNA Engine Opticon®2 cycler MJ Research, Waltham, MA, USA), gradually cooling from 99°C to 16°C over 70 min. Ligation was performed in 20 µl reaction volume using 1 unit of T4 DNA ligase (Invitrogen, Karlsruhe, Germany) and 100 ng of vector DNA in a 1:4 molar vector-insert ratio and incubated at 16 °C over night. 2 µl of ligation mixture was used to transform 40 µl competent TOP10 cells (Invitrogen, Karlsruhe, Germany) using standard electroporation procedure. The transformed cells were recovered in 0.8 ml of LB for 1 h at 37°C and plated on ampicillin (100 µg/ml) containing agar plates. Plasmid DNA preparations were made from over-night cultures of individual colonies using the Pure Yield® Plasmid MidiPrep System (Promega, Mannheim, Germany) following the manufacturer’s protocol. The correct sequence was verified via standard cycle sequencing for each shRNA cassette. Lentiviruses were made by transfecting packaging cells (HEK293T) with a 3-plasmid system. DNA for transfections was prepared by mixing 12 µg pCMVΔRR8.2, 1 µg pHIT G and 12 µg pLKO.1 plasmid DNA

with 62 µl of 2M $CaCl_2$ in a final volume of 500 µl. Subsequently 500 µl of 2x HBS phosphate buffer was drop wise added to the mixture and incubated for 10 min at RT. The 1 ml transfection mixture was added to 50% confluent HEK293T cell seeded the day before into a 10 cm well plate. Cells were incubated for 16 h (37 °C and 10% CO_2), and the media was changed to remove remaining transfection reagent. Lentiviral supernatants were collected at 36 h post-transfection and for each infection 3 ml supernatant containing 4 µg/ml polybrene was immediately used to infect target cells seeded the day before in 6 cm well plates to reach 70% confluency on the day of infection. Cells were incubated for 24 h, and the media was changed to remove virus particles. To control infection rate a parallel infection under the identical conditions targeting the same cell line was prepared using a lentiviral GFP expression control vector (pRRLU6-CPPT-pSK-GFP, kindly provided by Sheila Stewart). 6 days after infection 2 µg/ml puromycin was added to the cell culture media. shRNA oligonucleotide sequences were: **KIAA1199-6640s**: CCGG CCA GGA ATG TTG AAT GTC TTT CTC GAG AAA GAC ATT CAA CAT TCC TGG TTTTTG; **KIAA1199-6640as**: AATT CAAAAA CCA GGA ATG TTG AAT GTC TTT CTC GAG AAA GAC ATT CAA CAT TCC TGG; **KIAA1199-2396s:** CCGG CCC AGG TTA TTC AGA GCA CAT CTC GAG ATG TGC TCT GAA TAA CCT GGG TTTTTG; **KIAA1199-2396as**: AATT CAAAAA CCC AGG TTA TTC AGA GCA CAT CTC GAG ATG TGC TCT GAA TAA CCT GGG; **KIAA1199-1347s**: CCGG GCC ACT ACA ATG GAT GGA GTT CTC GAG AAC TCC ATC CAT TGT AGT GGC TTTTTG; **KIAA1199-1347as**: AATT CAAAAA GCC ACT ACA ATG GAT GGA GTT CTC GAG AAC TCC ATC CAT TGT AGT GGC; **KIAA1199-3302s**: CCGG GCG AAT GAA GAT CAT CAA GAA CTC GAG TTC TTG ATG ATC TTC ATT CGC TTTTTG; **KIAA1199-3302as**: AATT CAAAAA GCG AAT GAA GAT CAT CAA GAA CTC GAG TTC TTG ATG ATC TTC ATT CGC; **KIAA1199-3303s**: CCGG CGA ATG AAG ATC ATC AAG AAT CTC GAG ATT CTT GAT GAT

CTT CAT TCG TTTTTG; **KIAA1199-3303as**: AATT CAAAAA CGA ATG AAG ATC ATC AAG AAT CTC GAG ATT CTT GAT GAT CTT CAT TCG

Genotype of cell lines used for functional analyses had the following genotype: SW480 cells (MSS, wildtype K-Ras, mutated in TGFbeta, SMAD4 and TP53) and HCT116 cells (MSI, functional TGFbeta and TP53, mutated in CTNNB1, CDKN2A, MLH1, PIK3CA, KRas).

Microarray analyses were performed on cell lines manipulated as follows: i) SW480 cells constitutively expressing SMAD4 (D20) compared to control cells with an empty vector (K6). ii) LS174 cells with active Wnt-signaling and doxyrubicin treated inducing overexpression of an inactive TCF4 resulting in cells with a silenced Wnt signaling pathway as described previously (Andersen *et al.*, 2007;Schepeler *et al.*, 2007) iii) HCT116 (MSI) and SW480 (MSS) colon cancer cells were transiently transfected with a pCR3.1 mock or a pCR3.1:KIAA1199-V5-HIS-tag or GFP-tag plasmid using Lipofectamin (Invitrogen) according to the manufacturers description. Cells were harvested 24-48 h posttransfection and protein expression was monitored by Western blotting. iv) Lentiviral mediated delivery of shRNA sequences targeting KIAA1199 were applied to successfully knock-down KIAA1199 and SW480 pLKO sh-KIAA1199-3303 was compared to a control vector.

Quantitative real-time PCR (qRT-PCR) Total RNA from cell lines was isolated by acid phenol extraction. cDNA was synthesized using 2 µg of total RNA, oligo(dT)$_{18}$ primers and SuperScript^{TM} II RNase H^{-} reverse transcriptase (Invitrogen, Karlsruhe, Germany) following the manufacturer's protocol and diluted to a final volume of 50 µl with 1x first strand buffer. Intron spanning primer sets for qRT-PCR were designed using Primer Express 2.0 software (Applied Biosystems, Foster City, CA, USA) (KIAA1199-3367-S: GGAGCACCCATTACCAGCAA and KIAA1199-3489-AS:

AGTCGCCCTTGTTGAAGTTGA). qRT-PCR was performed using a SYBR Green I reaction mixture containing 75 mM Tris-HCl (pH 8.8), 20 mM ammonium sulfate, 0.01 % (v/v) Tween 20, 2 mM magnesium chloride (all Sigma-Aldrich, Munich, Germany), 1 µl of a 600-fold dilution of SYBR Green I (BioWhittaker, Rockland, ME, USA), 2.5 U *Taq* polymerase (NEB, Frankfurt a.M., Germany), 0.2 mM dNTP (Promega, Mannheim, Germany) and 0.2 µM of forward and reverse primer (QIAgen, Hilden, Germany) in a final reaction volume of 20 µl. Reactions were run on a DNA Engine Opticon®2 cycler (MJ Research, Waltham, MA, USA). The cycling conditions consisted of 3 min initial denaturation at 94°C and 40 cycles of 94°C for 30 sec, 60°C for 30 sec, 72°C for 30 sec and 80°C for 3 sec. Fluorescence was measured at the last step of each cycle. Melting curves were obtained after each PCR run and showed single PCR products. cDNAs were run in triplicate, non-RT (without reverse transcriptase) and no-template controls were run in duplicates. PCR efficiencies were determined using serial dilutions of a cDNA derived from cell line SW480 Expression levels for genes of interest and for housekeeping genes were measured for in independent PCR runs. Expression ratios were calculated using the geometric mean expression of the housekeeping genes GAPD, HPRT1 and PPIA to normalize the expression data for the gene of interest.

Supplementary References

Andersen CL, Schepeler T, Thorsen K, Birkenkamp-Demtroder K, Mansilla F, Aaltonen LA, Laurberg S, Orntoft TF (2007) Clusterin expression in normal mucosa and colorectal cancer. *Mol Cell Proteomics* **6** (6): 1039-1048

Cartharius K, Frech K, Grote K, Klocke B, Haltmeier M, Klingenhoff A, Frisch M, Bayerlein M, Werner T (2005) MatInspector and beyond: promoter analysis based on transcription factor binding sites. *Bioinformatics* **21** (13): 2933-2942

Fink SP, Kariv R, Platzer P, Xin B, Mikkola D, Nosrati AYM, Willson JKV, Willis J, Dawson D, Wilson K, Markowitz SD (2006) Colon Cancer Secreted Protein-1 (CCSP-1), A Novel Candidate Serological Marker of Colon Neoplasia. *Abstract presented at 4th Annual Protein Biomarkers 2006 Meeting, Philadelphia*

Mansilla F, Birkenkamp-Demtroder K, Kruhoffer M, Sorensen FB, Andersen CL, Laiho P, Aaltonen LA, Verspaget HW, Orntoft TF (2007) Differential expression of DHHC9 in microsatellite stable and instable human colorectal cancer subgroups. *British J Cancer E-Pub ahead* 10.1038/sj.bjc.6603818 [doi]

Mathivanan S, Simpson RJ (2009) ExoCarta: A compendium of exosomal proteins and RNA. *Proteomics* **9** (21): 4997-5000

Øster B, Thorsen K, Lamy P, wojdacz TK, hansen LL, Birkenkamp-Demtroder K, Sørensen KD, Laurberg S, Orntoft TF, Andersen C (2011) Identification and validation of highly frequent CpG island hypermethylation in colorectal adenomas and carcinomas. *Int J Cancer in press*

Schepeler T, Mansilla F, Christensen LL, Orntoft TF, Andersen CL (2007) Clusterin expression can be modulated by changes in TCF1-mediated Wnt signaling. *J Mol Signal* **2:6.** 6

Stewart SA, Dykxhoorn DM, Palliser D, Mizuno H, Yu EY, An DS, Sabatini DM, Chen IS, Hahn WC, Sharp PA, Weinberg RA, Novina CD (2003) Lentivirus-delivered stable gene silencing by RNAi in primary cells. *RNA* **9** (4): 493-501
